# Adaptive Rewiring of Nucleotide Metabolism in Therapeutic Resistance

**DOI:** 10.3390/cancers18213498

**Published:** 2026-10-30

**Authors:** Bengisu Dayanc, Ganfei Xu, Xiaoshu Zhou, Jinpeng Chen, Noah Mueller, Harsimran Kaur, Breanna Pham, Satoshi Yoda, Jaime L. Schneider

**Affiliations:** 1 Department of Medical Oncology, Division of Molecular and Cellular Oncology, Dana-Farber Cancer Institute, Boston, MA 02115, USA; bengisu_uluatadayanc@dfci.harvard.edu (B.D.);; 2 Key Laboratory of Chinese Medicine Resource and Compound Prescription, Ministry of Education, Hubei University of Chinese Medicine, Wuhan 430065, China; 3 Department of Medicine, Brigham and Women’s Hospital, Harvard Medical School, Boston, MA 02115, USA

**Keywords:** metabolic reprogramming, nucleotide metabolism, acquired therapeutic resistance, immunotherapy resistance, targeted therapy resistance

## Abstract

Therapeutic resistance remains a major barrier to durable cancer control and is increasingly recognized as a dynamic process shaped not only by genetic evolution but also by metabolic adaptation. Nucleotide metabolism is uniquely positioned at this interface, supporting DNA and RNA synthesis while also regulating signaling, energy homeostasis, redox balance, genome maintenance, and cellular stress responses. In this Review, we examine emerging evidence that cancer cells in solid tumors adapt nucleotide metabolism in response to therapeutic pressure through coordinated remodeling of de novo biosynthesis, nucleotide salvage, metabolite transport, and pathway-specific feedback mechanisms. We discuss how cytotoxic chemotherapy, targeted therapies, immunotherapies, and radiation impose distinct but convergent pressures on nucleotide homeostasis and how restoration or redistribution of nucleotide pools can promote survival, DNA repair, persistent oncogenic signaling, and immune evasion. We further highlight the reciprocal relationship between oncogenic signaling and nucleotide metabolism, as well as the contribution of tumor suppressor loss and the tumor microenvironment to context-specific metabolic dependencies. Rather than functioning only as a biosynthetic requirement of proliferating cells, nucleotide metabolism emerges as a flexible stress-response network that can facilitate adaptation to cancer therapy. Defining the mechanisms that maintain nucleotide balance in resistant tumors may reveal therapeutically actionable dependencies and inform rational combination strategies designed to prevent or overcome treatment resistance.

## Simple Summary

Resistance to anti-cancer therapy remains a persistent and universal challenge in cancer treatment. Even the most effective targeted therapies are often unable to achieve a cure, as cancer cells learn to adapt and develop resistance. Despite major advances in tumor genome sequencing, we frequently cannot identify the mechanisms responsible for treatment resistance through genetic changes alone. An emerging area of research focuses on how cancer cells reprogram their metabolism to survive the stress imposed by anti-cancer therapies. In particular, nucleotide metabolism, which supports the production of the building blocks needed for DNA and RNA synthesis, plays essential roles in energy balance, signaling, and the repair of damaged DNA. Increasing evidence suggests that cancer cells can alter how they produce, use, and recycle nucleotides to survive treatment and sustain tumor growth. In this Review, we summarize emerging evidence linking changes in nucleotide metabolism to resistance across multiple cancer treatment modalities, including chemotherapy, targeted therapy, immunotherapy, and radiation. We also discuss how these metabolic adaptations interact with cancer-driving genetic alterations and the surrounding tumor environment. By highlighting nucleotide metabolism as an important but often overlooked mechanism of therapeutic resistance, we aim to identify new opportunities for developing treatment strategies that may prevent or overcome resistance and ultimately improve outcomes for patients with cancer.

## 1. Introduction

Cancer cells undergo metabolic reprogramming to sustain proliferation and adapt to therapeutic pressure, establishing metabolic plasticity as a central hallmark of cancer [1,2]. Although early studies dating back more than a century emphasized aerobic glycolysis as a metabolic mechanism exploited by cancer cells [3], it is now clear that tumors dynamically rewire multiple metabolic networks, including central carbon metabolism, amino acid utilization, nucleotide synthesis, and redox homeostasis. Importantly, this reprogramming is not limited to de novo tumorigenesis. Metabolic rewiring can also serve as a critical determinant of therapeutic response, enabling cancer cells to survive and evolve under treatment pressure. Cytotoxic chemotherapy, targeted therapies, immunotherapies, and radiation each impose distinct metabolic stresses that reshape the substrates and energetic resources required for DNA replication and repair, transcriptional remodeling, and cell-fate decisions. Accordingly, metabolic pathways function not as static biosynthetic programs but as adaptable networks that integrate oncogenic signaling, cellular stress responses, microenvironmental constraints, and therapeutic pressure [4].

Nucleotides are best recognized as the building blocks of DNA and RNA, but their cellular functions extend far beyond nucleic acid synthesis. Purine and pyrimidine metabolites support energy transfer, cofactor generation, intracellular signaling, protein translation, and stress adaptation [5]. Cancer cells therefore co-opt nucleotide metabolism not only to meet increased biosynthetic demand but also to sustain signaling and homeostasis under fluctuating environmental and therapeutic conditions. This broader framework is especially relevant in resistant disease, where nucleotide metabolic programs can be remodeled rather than simply upregulated. Changes in de novo synthesis, salvage, transport, and nucleotide interconversion can preserve nucleotide availability despite therapeutic stress, while imbalance among individual nucleotide pools can itself alter signaling fidelity, replication dynamics, and genome stability [6]. Thus, nucleotide metabolism is increasingly understood as a regulatory hub linking proliferative demand with adaptive stress responses.

Lung cancers provide a particularly informative context in which to examine these dependencies, as high proliferative demand and recurrent oncogenic signaling can create sensitivity to perturbations in nucleotide [7]. Oncogenic drivers common in lung cancer, including KRAS, ALK, and EGFR alterations, increase flux through nucleotide biosynthetic pathways while also increasing sensitivity to nucleotide imbalance and replication stress [8]. Large-scale genomic and functional datasets further identify copy-number gains, transcriptional upregulation, and dependency signals involving nucleotide metabolic enzymes across lung cancer subtypes, consistent with selective pressure to sustain nucleotide homeostasis [9]. These observations suggest that the requirement to maintain nucleotide homeostasis creates context-dependent therapeutic liabilities that may be unmasked during treatment, particularly when compensatory pathways are constrained.

This Review examines therapeutic resistance through the lens of nucleotide metabolism, with an emphasis on the dynamic balance between de novo synthesis and salvage pathways and the feedback mechanisms that enable compensation under stress. Rather than viewing nucleotide metabolism as only a constitutive biosynthetic requirement, we frame it as an adaptive stress-response network that is remodeled by cancer therapy. We highlight how traditional cytotoxic chemotherapy, targeted therapies, immunotherapies, and radiation impose distinct but sometimes convergent pressures on nucleotide homeostasis, and how cancer cells respond through pathway plasticity, altered signaling, microenvironmental interactions, and mechanisms that preserve nucleotide balance. Finally, we synthesize emerging strategies to exploit context-specific nucleotide metabolic dependencies, with the goal of identifying rational approaches to prevent or overcome therapeutic resistance.

## 2. Nucleotide Metabolism in Cancer

### 2.1. Purine Nucleotide Regulation

**Figure 1:**
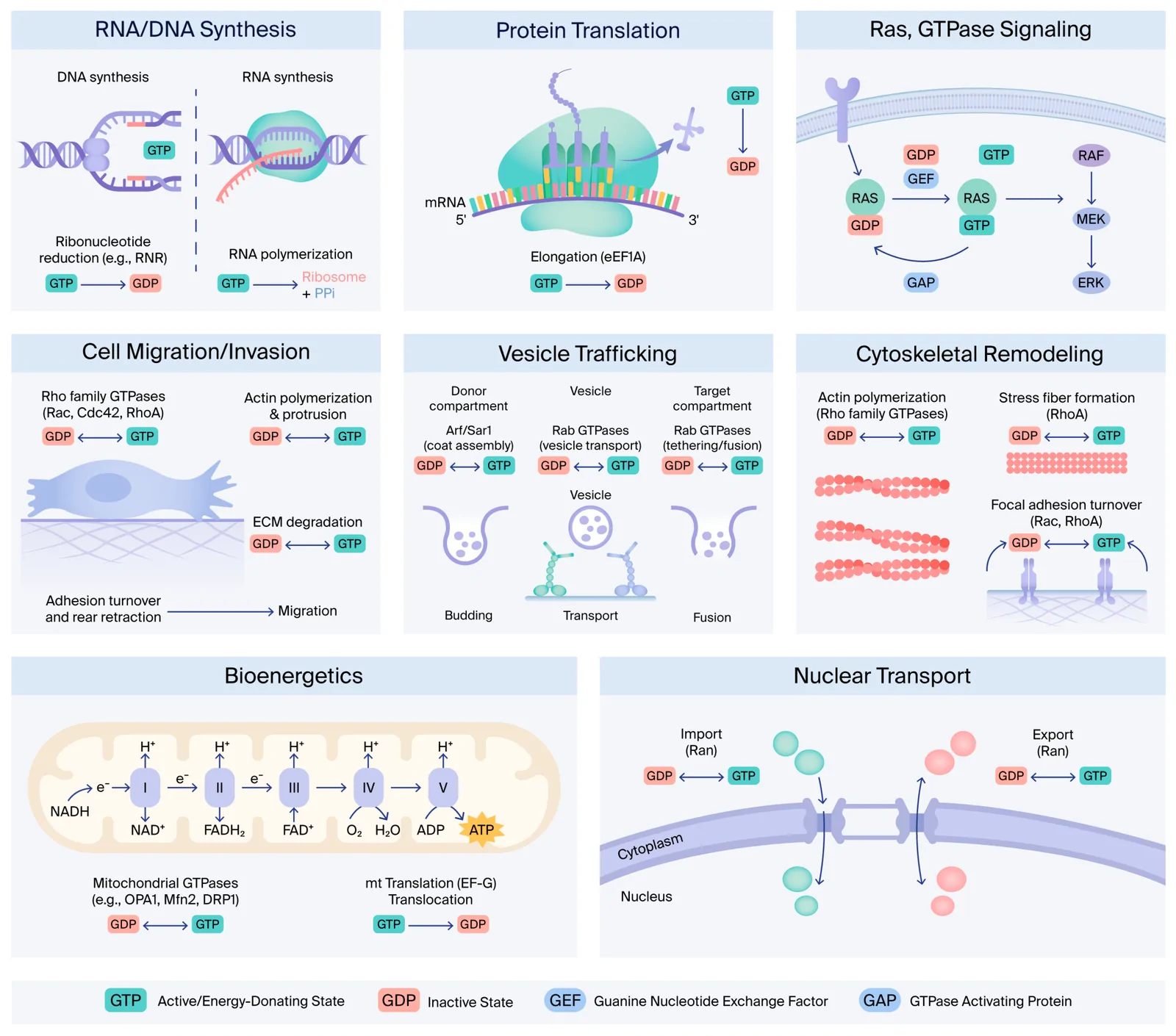
**Diverse cellular functions of guanine nucleotides.** GTP and GDP cycling supports multiple fundamental cellular processes beyond nucleic acid synthesis. GTP is required for RNA and DNA synthesis, protein translation, G protein-family signaling, cell migration and invasion, vesicle trafficking, cytoskeletal remodeling, mitochondrial bioenergetics, and nuclear transport. Abbreviations: ADP/ATP, adenosine di-/triphosphate; ECM, extracellular matrix; eEF1A, eukaryotic elongation factor 1A; EF-G, elongation factor G; GAP, GTPase-activating protein; GDP/GTP, guanosine di-/triphosphate; GEF, guanine nucleotide exchange factor; MEK, MAPK/ERK kinase; NAD^+^/NADH, oxidized/reduced nicotinamide adenine dinucleotide; FAD/FADH_2_, oxidized/reduced flavin adenine dinucleotide; PPi, inorganic pyrophosphate; RNR, ribonucleotide reductase.

Beyond serving as substrates for RNA and DNA synthesis, purine metabolites are essential for energy homeostasis, intracellular signaling, protein translation, mitochondrial metabolism, and cofactor production, supporting a wide range of cellular programs required for survival and proliferation [10] (Figure 1). Distal purine nucleotides such as ATP and GTP function in bioenergetics and signal transduction, while purine-derived moieties can serve as structural backbones for several essential metabolic cofactors including NAD, FAD, and coenzyme A.

Purine homeostasis is maintained through a coordinated balance between de novo synthesis and salvage pathways. In most cell types, a large fraction of intracellular purine nucleotides is replenished through salvage mechanisms that recycle purine bases generated by nucleic acid turnover [11,12]. Hypoxanthine-guanine phosphoribosyltransferase (HPRT) catalyzes the salvage of hypoxanthine and guanine to inosine monophosphate (IMP) and guanosine monophosphate (GMP), respectively [13], whereas adenine phosphoribosyltransferase (APRT) converts adenine to adenosine monophosphate (AMP). When purine demand exceeds salvage capacity, flux through the de novo pathway can increase to maintain nucleotide sufficiency. Extracellular purines can provide an additional source of substrate through cellular transport systems, although how these transport processes are regulated and which metabolites predominate in specific tumor contexts remain active areas of investigation.

Although rapidly proliferating cells were traditionally thought to depend predominantly on de novo purine synthesis, recent evidence indicates that salvage metabolism can also make a major contribution in tumors. Tran et al. demonstrated that adenine and inosine are the predominant circulating purine precursors supporting nucleotide synthesis in both normal tissues and tumors, whereas hypoxanthine contributes minimally *in vivo* [14]. Consistent with this concept, Tabata et al. identified HPRT1-dependent purine salvage as a critical determinant of proliferation in a subset of small cell lung cancer (SCLC) [15]. SCLC cells dynamically utilize both salvage and de novo pathways, illustrating the metabolic plasticity available to sustain purine pools. Metabolomic profiling of HPRT1-knockout cells revealed accumulation of pentose phosphate pathway intermediates accompanied by increased flux through de novo purine synthesis, providing evidence for compensatory cross-talk between these pathways to preserve nucleotide homeostasis.

Guanosine triphosphate (GTP) has emerged as an important regulator of cancer cell proliferation and signaling. A major determinant of guanine nucleotide production is inosine monophosphate dehydrogenase 2 (IMPDH2), a rate-limiting enzyme in de novo guanine nucleotide synthesis [16] that catalyzes the NAD^+^-dependent conversion of inosine monophosphate (IMP) to xanthosine monophosphate (XMP) [17]. Increased GTP availability can support oncogenic processes including proliferation, migration, and therapeutic resistance through modulation of GTP-dependent signaling pathways [18]. Accordingly, IMPDH inhibitors such as mycophenolate mofetil (MMF) and mycophenolic acid (MPA) have gained interest as potential anticancer therapies [19,20]. MYC-high/ASCL1-low small cell lung cancers (SCLC), for instance, are highly dependent on IMPDH activity to maintain guanine nucleotide pools [21]. MYC can reinforce this state by transcriptionally upregulating IMPDH1 and IMPDH2 as part of a broader purine metabolic program [22,23]. These tumors exhibit enhanced guanine nucleotide biosynthesis and increased sensitivity to IMPDH inhibition, which suppresses ribosomal RNA synthesis and tumor growth. Although these findings identify IMPDH as a metabolic vulnerability in lung cancer, compensatory salvage pathways may bypass upstream blockade of de novo guanine nucleotide synthesis and limit the durability of this approach.

At the convergence of de novo and salvage guanine nucleotide metabolism, guanylate kinase 1 (GUK1) catalyzes the phosphorylation of GMP to GDP and thereby contributes to maintenance of the cellular GTP pool [18]. In oncogene-driven lung cancers, fusion tyrosine kinases including ALK, ROS1, and RET can influence guanine nucleotide availability through direct regulation of metabolic signaling nodes. Phosphorylation-mediated regulation of GUK1 has been proposed to increase GDP and GTP pools, facilitating RAS-GTP loading and amplifying MAPK signaling activity. In parallel, guanosine monophosphate synthetase (GMPS), a key enzyme in the de novo pathway, catalyzes the conversion of xanthosine monophosphate (XMP) to GMP, supporting sustained guanine nucleotide production in proliferating cancer cells [24].

Emerging evidence supports a bidirectional relationship between purine metabolism and oncogenic signaling. Elevated intracellular GTP can enhance the activity of small GTPases such as RAS and RAC, promoting downstream processes including MAPK signaling and cytoskeletal remodeling [25]. Conversely, oncogenic signaling can directly regulate purine biosynthesis. For example, Wnt/β-catenin signaling induces IMPDH2 expression, establishing a feedforward loop that promotes tumor growth and contributes to therapeutic resistance in oxaliplatin-resistant colorectal cancer [26]. MYC-driven tumors similarly exhibit coordinated upregulation of multiple enzymes in the purine biosynthetic network. This reciprocal regulation positions purine metabolism not only as an output of oncogenic signaling, but also as a potential amplifier of signaling states required for sustained proliferation.

### 2.2. Pyrimidine Nucleotide Regulation

Pyrimidine nucleotides are synthesized through a conserved pathway that couples nutrient availability to proliferative demand. Unlike purine biosynthesis, the pyrimidine ring is assembled before conjugation to ribose-5-phosphate. The first three enzymatic reactions are catalyzed by CAD, a multifunctional protein containing carbamoyl phosphate synthetase 2, aspartate transcarbamylase, and dihydroorotase activities. CAD therefore represents a major regulatory node and is controlled by growth-signaling inputs, including mTORC1, that coordinate anabolic signaling with pyrimidine synthesis [27,28]. Dihydroorotate is subsequently oxidized to orotate by dihydroorotate dehydrogenase (DHODH), an enzyme embedded in the inner mitochondrial membrane that directly links de novo pyrimidine synthesis to mitochondrial electron transport [29]. Beyond its biosynthetic role, DHODH also protects against mitochondrial ferroptosis by maintaining reduced ubiquinone pools, identifying a therapeutic vulnerability in GPX4-low tumors [30]. Downstream reactions generate uridine monophosphate (UMP), the precursor for pyrimidine nucleotide pools required for RNA and DNA synthesis. Further regulation also occurs through CTP synthetases (CTPS1/2), which catalyze the conversion of UTP to CTP. Reduced CTPS2 expression has been associated with resistance to 5-FU in colorectal cancer, linking pyrimidine nucleotide balance to chemotherapy sensitivity [31].

Studies have also linked energy-sensing pathways to the regulation of pyrimidine metabolism, helping match nucleotide production to cellular energetic and redox state. The tumor suppressor kinase LKB1 (STK11) is a central regulator of energy homeostasis, primarily through activation of AMP-activated protein kinase (AMPK). Loss of LKB1 alters metabolic flux and can increase reliance on de novo pyrimidine biosynthesis [28]. In this setting, mitochondrial dysfunction can increase dependence on DHODH-mediated dihydroorotate oxidation, while reduced AMPK activity relaxes metabolic checkpoints that normally constrain anabolic biosynthesis. This rewiring creates a state in which LKB1-deficient tumors become selectively vulnerable to DHODH inhibition [27], highlighting nucleotide metabolism as a potential therapeutic liability in this molecular subset of lung cancer. Pyrimidine nucleotide deaminases provide another layer of regulation with therapeutic implications. The recently characterized dCTP deaminase CDADC1 can inactivate the active triphosphate metabolites of gemcitabine and decitabine, reducing drug sensitivity, whereas dCMP deaminase (DCTD) has distinct effects on nucleotide metabolism and can promote sensitivity to these agents [32].

Pyrimidine salvage also contributes to maintenance of intracellular nucleotide pools by recycling nucleosides derived from extracellular sources or intracellular turnover. Cellular nucleoside uptake is mediated by two major classes of transporters: equilibrative nucleoside transporters (ENTs; SLC29 family) and concentrative nucleoside transporters (CNTs; SLC28 family). ENTs are bidirectional passive transporters that facilitate nucleoside flux along concentration gradients, whereas CNTs mediate sodium-dependent nucleoside import [29]. Once internalized, nucleosides such as uridine and cytidine are phosphorylated by uridine-cytidine kinases to regenerate nucleotide monophosphates and replenish intracellular pyrimidine pools. Studies have demonstrated that mTORC1 promotes pyrimidine salvage by stabilizing UCK2 through suppression of CTLH-WDR26-mediated degradation, linking growth signaling to nucleotide recycling and sensitivity to pyrimidine analog therapies [33]. Importantly, the relative contributions of salvage and de novo synthesis can shift with cellular state or therapeutic pressure. Increased reliance on salvage has been observed following DHODH inhibition, underscoring the metabolic flexibility that preserves pyrimidine homeostasis [34].

### 2.3. Nucleotide Compartmentalization and Organelle Crosstalk

Nucleotide metabolism is spatially organized, enabling inter-organelle communication and localized control of nucleotide utilization. Cytosolic and mitochondrial pathways contribute to cellular nucleotide supply, while nuclear demand is concentrated at sites of DNA replication and repair. This spatial organization helps couple DNA synthesis to nucleotide availability in real time and may limit the consequences of local substrate depletion. Inter-organelle crosstalk and subnuclear compartmentalization therefore provide an additional layer of regulation that integrates global metabolic supply with localized nucleotide demand.

In addition to nuclear compartmentalization, nucleotide pools are tightly regulated within mitochondria, which harbor their own genome and require a coordinated supply of nucleotides for mitochondrial DNA replication and function. Dedicated mitochondrial transporters, including SLC25A33 and SLC25A36, facilitate pyrimidine nucleotide transport across the inner mitochondrial membrane, linking cytosolic nucleotide availability to mitochondrial nucleic acid synthesis [35]. DHODH localizes to the outer surface of the inner mitochondrial membrane and uses ubiquinone as an electron acceptor [36,37]. Electrons transferred from DHODH reduce ubiquinone to ubiquinol, which is subsequently reoxidized by complex III within the mitochondrial respiratory chain. This biochemical coupling directly links pyrimidine biosynthesis to mitochondrial electron transport and energy metabolism [37,38]. Consequently, de novo pyrimidine synthesis depends on a functional respiratory chain and can be impaired by mitochondrial dysfunction. Conversely, inhibition of DHODH perturbs both pyrimidine production and cellular redox balance, increasing the vulnerability of GPX4-low tumors to ferroptosis [30,39].

Purine biosynthesis is also organized spatially. When cellular demand exceeds salvage capacity, de novo purine biosynthesis can be upregulated to meet increased nucleotide requirements [40]. Mitochondrial one-carbon metabolism provides an additional link between organelles, generating formate through the SHMT2-MTHFD2-MTHFD1L pathway to support cytosolic purine and thymidylate synthesis. Disruption of this mitochondrial pathway can trigger compensatory cytosolic one-carbon metabolism, highlighting metabolic plasticity across compartments [41]. Moreover, enzymes in purine synthesis can assemble into multienzyme complexes known as purinosomes, which localize near mitochondria and along microtubule networks to facilitate metabolic coordination [42]. These cytosolic assemblies bring multiple de novo purine biosynthetic enzymes into proximity, promoting substrate channeling and helping regulate flux toward AMP and GMP production [43,44]. Purinosomes also associate spatially with mitochondria, and mitochondrial dysfunction increases their abundance as a compensatory response [45]. This organization suggests reciprocal coupling between de novo purine synthesis and mitochondrial metabolism, allowing synthetic demand to be coordinated with local energy and substrate availability. Similarly, disruption of endoplasmic reticulum integrity through MTM1 knockout destabilizes purinosomes, impairs substrate channeling, and reduces intracellular purine nucleotide levels, illustrating how multiple organelles can influence nucleotide biosynthetic organization [46].

Beyond mitochondria and the endoplasmic reticulum, lysosomes coordinate nutrient availability with anabolic signaling through mTORC1. Lysosomal amino acid sensing, mediated in part by SLC38A9, regulates mTORC1 activation and thereby links nutrient availability to cellular growth and nucleotide biosynthetic demand [47]. This spatial organization reinforces a view of nucleotide metabolism as more than a housekeeping process required for proliferation. Instead, these metabolites and pathways form a coordinated network that interfaces with oncogenic signaling, genome maintenance, organelle function, and cellular stress adaptation across multiple compartments.

## 3. Chemotherapy-Induced Reprogramming of Nucleotide Metabolism

**Figure 2:**
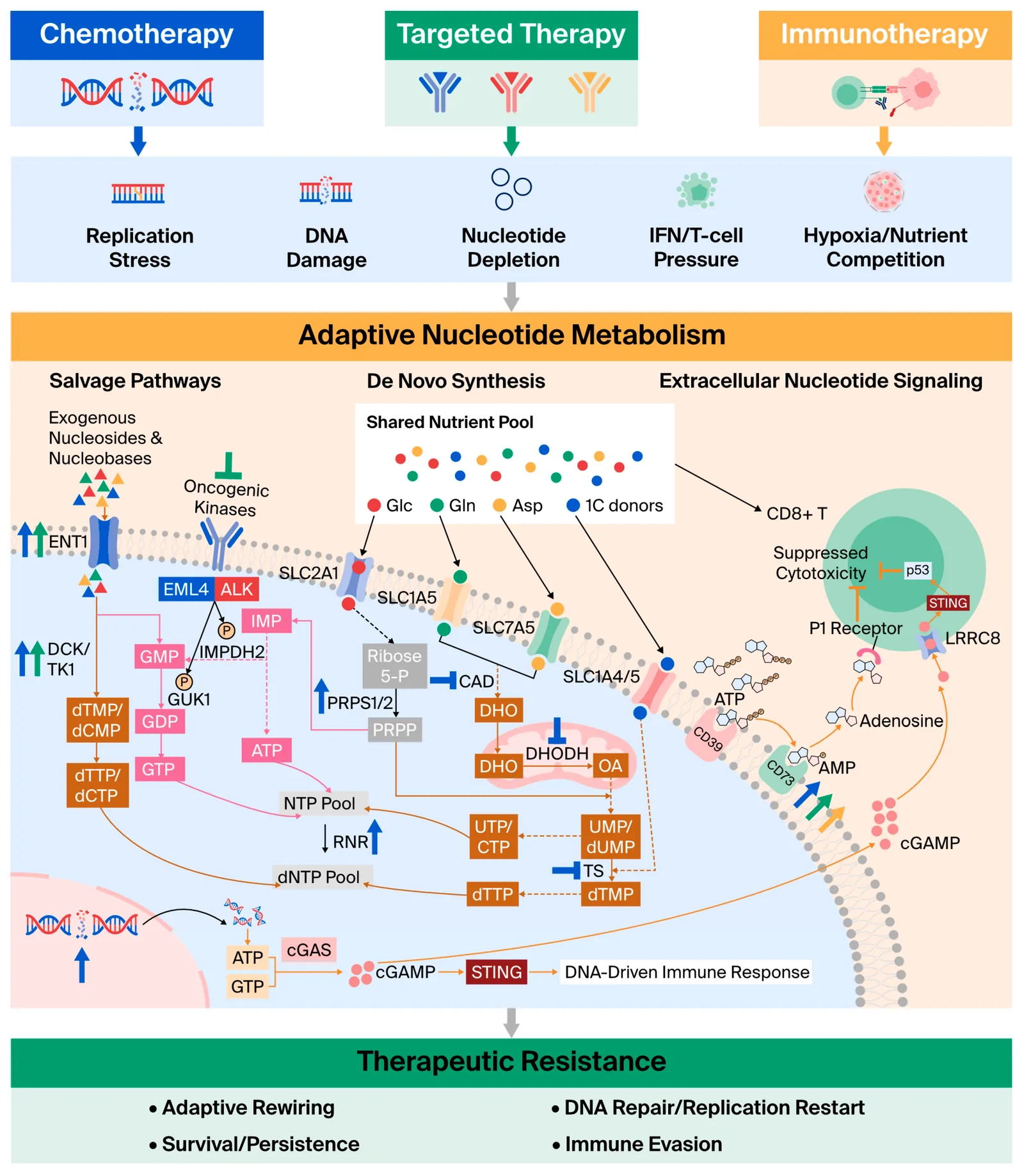
**Nucleotide metabolism rewiring as a shared adaptive axis across cancer therapies**. Chemotherapy, targeted therapy, and immunotherapy lead to distinct but sometimes convergent stresses on tumor cells and the tumor microenvironment. In response, cancers adapt through remodeling of nucleotide metabolism, including altered de novo synthesis, nucleoside salvage, transport, and extracellular nucleotide signaling. These programs help maintain nucleotide homeostasis, support DNA repair and replication restart, promote tumor-cell survival and persistence, and shape immune suppression within the tumor microenvironment, contributing to acquired therapeutic resistance. Arrows and colors: Solid arrows indicate direct enzymatic reactions or transport; dashed arrows, multistep or indirect processes; upward arrows, adaptive upregulation under therapeutic stress; and T-bars, inhibition. Blue, green, and orange denote chemotherapy-, targeted therapy-, and immunotherapy-induced effects, respectively. Purple and brown indicate purine and pyrimidine metabolism, respectively. Abbreviations: 1C, one-carbon; CAD, carbamoyl-phosphate synthetase 2/aspartate transcarbamylase/dihydroorotase; cGAS, cyclic GMP-AMP synthase; CD39/CD73, cluster of differentiation 39/73; DCK, deoxycytidine kinase; DHODH, dihydroorotate dehydrogenase; EML4-ALK, echinoderm microtubule-associated protein-like 4–anaplastic lymphoma kinase; ENT1, equilibrative nucleoside transporter 1; Glc, glucose; Gln, glutamine; GUK1, guanylate kinase 1; IMPDH2, inosine monophosphate dehydrogenase 2; LRRC8, volume-regulated anion channel; PRPS1/2, phosphoribosyl pyrophosphate synthetase 1/2; RNR, ribonucleotide reductase; SLC, solute carrier transporters; STING, stimulator of interferon genes; TK1, thymidine kinase 1; TS, thymidylate synthase.

Chemotherapy has been used in systemic cancer treatment for more than seven decades, beginning with nitrogen mustards and antifolates [48]. Despite advances in targeted and immunotherapeutic approaches, chemotherapy remains a critical component across solid tumors and hematologic malignancies [49,50]. More than 20 approved nucleotide and nucleotide analogs are used in cancer therapy and account for nearly 20% of drugs in cancer treatment [51]. These agents perturb nucleotide homeostasis by depleting endogenous nucleotide pools, mimicking physiological nucleotides, or inducing DNA damage that acutely increases nucleotide requirements for replication and repair (Figure 2).

Clinically, incomplete tumor eradication remains common, as illustrated for instance by the low pathologic complete response rate with neoadjuvant chemotherapy in CheckMate 816 [52]. Cancer cells can respond by rewiring nucleotide synthesis, salvage, and utilization to restore nucleotide balance and survive treatment. Here, we focus on these tumor-intrinsic adaptive responses as mechanisms of chemotherapy tolerance and resistance.

### 3.1. Replication Stress, DNA Damage, and Nucleotide Imbalance

Many cytotoxic agents induce replication stress by directly perturbing nucleotide pools, interfering with DNA synthesis, or generating lesions that obstruct replication fork progression. Platinum agents form intra- and interstrand DNA crosslinks that distort DNA structure and can promote replication fork stalling and collapse, double-strand breaks (DSBs), and accumulation of single-stranded DNA (ssDNA) [53,54]. Antimetabolites such as 5-Fluorouracil (5-FU) and Gemcitabine act more directly on nucleotide metabolism and DNA synthesis: 5-FU inhibits thymidylate synthase, resulting in dTMP depletion and nucleotide pool imbalance, whereas gemcitabine incorporation into DNA causes chain termination and impaired elongation [55,56]. Topoisomerase inhibitors such as Etoposide stabilize topoisomerase-DNA cleavage complexes; during S phase, collision of replication forks with these complexes converts transient lesions into persistent DNA breaks [57].

To survive replication stress, cancer cells activate a coordinated DNA damage response (DDR) that couples cell-cycle control and DNA repair with metabolic adaptation. Replication-associated ssDNA primarily activates ATR–CHK1 signaling, whereas fork collapse and DSB formation activate ATM; downstream effectors including CHK1, CHK2, and p53 regulate cell-cycle checkpoints, replication fork stability, and DNA repair [58]. Importantly, nucleotide imbalance is itself a source of replication stress: Diehl et al. showed that both nucleotide depletion and imbalances between individual nucleotide species impair S-phase progression and activate ATR-dependent signaling [6].

DDR pathways also help preserve nucleotide availability under stress. ATR-CHK1 signaling can sustain intracellular dNTP pools through regulation of ribonucleotide reductase (RNR), supporting continued DNA replication and repair [59,60,61,62]. In parallel, p53 functions as a metabolic checkpoint that modulates nucleotide homeostasis, in part through regulation of the pentose phosphate pathway (PPP) [61,63]. This reciprocal coupling between nucleotide homeostasis and the DDR may increase dependence on replication-stress response pathways and provides a rationale for combining ATR–CHK1 inhibition with replication stress-inducing chemotherapy.

### 3.2. Chemotherapy-Induced Metabolic Compensation

Nucleotide biosynthesis can be further induced in response to chemotherapy-mediated nucleotide depletion [64,65]. Increased de novo synthesis can help meet the acute nucleotide demands of DNA repair and replication fork restart [64,65]. For instance, 5-FU-mediated thymidylate synthase inhibition depletes dTTP and elicits compensatory responses that sustain DNA synthesis in colorectal cancer [56], whereas antifolate agents such as methotrexate disrupt one-carbon metabolism and nucleotide biosynthesis [56]. mTORC1-mediated activation of CAD provides another route to increase de novo pyrimidine synthesis and restore nucleotide pools under therapeutic stress [66].

### 3.3. Nucleotide Salvage as a Compensatory Pathway

When de novo synthesis is impaired or insufficient, cancer cells can increase reliance on nucleoside uptake and salvage to maintain nucleotide homeostasis [67]. Salvage pathways provide an energetically favorable route to replenish nucleotide pools and can therefore buffer chemotherapy-induced nucleotide stress. Tumor cells can increase nucleoside uptake through altered transporter expression or activity [68]. Exogenous thymidine, for example, can bypass thymidylate synthesis blockade by 5-FU or methotrexate, restoring dTTP pools and DNA synthesis [56,69]. Broader nucleoside supplementation can also reduce replication stress and DNA damage by replenishing intracellular dNTP pools [63]. Nucleoside transporters such as ENT1 (SLC29A1) and salvage enzymes including deoxycytidine kinase (DCK) and thymidine kinase 1 (TK1) support this adaptive capacity. Thus, salvage-dependent buffering of nucleotide imbalance may both promote chemotherapy tolerance and create a context-specific metabolic vulnerability [70,71].

## 4. Nucleotide Adaptations in Acquired Resistance to Targeted Therapies

### 4.1. Oncogene-Driven Rewiring of Nucleotide Metabolism

Next-generation sequencing (NGS) has enabled molecular stratification of cancers and dramatically expanded the use of genotype-directed therapy, supporting the development of tumor-agnostic treatment strategies defined by molecular alterations rather than tissue of origin [72]. Lung cancer provides an instructive model for interrogating oncogenic signaling and metabolism, with clinically actionable drivers spanning receptor tyrosine kinases (RTKs), including EGFR, MET, and HER2; oncogenic fusions involving ALK, ROS1, and RET; and alterations within the RAS-MAPK pathway [73]. Beyond their canonical signaling functions, these oncogenic drivers regulate metabolic programs that sustain nucleotide production. Consequently, targeted inhibition can create an acute metabolic constraint that tumor cells must overcome, making nucleotide homeostasis an important component of both drug tolerance and acquired resistance.

RTK signaling can directly couple oncogene activity to nucleotide biosynthesis. In lung adenocarcinoma, EGFR signaling supports glycolytic and pentose phosphate pathway (PPP) flux, thereby maintaining carbon substrates required for nucleotide production. Pharmacologic EGFR inhibition disrupts this metabolic state; erlotinib rapidly reduces multiple glycolytic and PPP intermediates, including FBP, DHAP, 3PG, PEP, lactate, and 6PG [74]. EGFR also directly regulates de novo pyrimidine synthesis through phosphorylation of CAD at Ser1859 [74]. Importantly, however, a subset of EGFR-mutant cells persists despite pathway inhibition and enters a drug-tolerant state associated with subsequent relapse [75]. These persister cells exhibit increased NRF2 activity and enhanced purine and pyrimidine nucleotide synthesis [76], suggesting that restoration of nucleotide supply may support survival during the early phases of therapeutic adaptation.

Similar signaling-metabolic coupling is observed in other EGFR- and MET-driven tumors. EGFR-altered glioblastomas show increased expression of and dependence on pyrimidine biosynthetic enzymes, including CAD and DHODH [77]. EGFR activation and amplification promote phosphorylation of CAD at S1859 and T456, respectively [77]. MET signaling also regulates de novo purine synthesis: MET inhibition depletes PRPP and FGAR and reduces expression of enzymes across the purine biosynthetic pathway [78]. This transcriptional suppression is accompanied by reduced expression of purine biosynthetic enzymes following MET inhibition [78]. These observations support a model in which oncogenic RTKs establish nucleotide-producing states that are disrupted, but not necessarily abolished, by targeted therapy.

Oncogenic RTK fusions provide a direct example of this relationship. RTK fusions are detected across solid tumors, with relatively high frequencies in thyroid and lung cancers [79]. Rearrangements involving ALK, RET, ROS1, FGFR, EGFR/ErbB or MET generate constitutively active kinases that signal through pathways including MAPK and PI3K [79]. These downstream pathways promote PRPP production and de novo nucleotide synthesis, thereby supporting intracellular nucleotide pools required for proliferation [80,81]. Emerging data further suggest that fusion kinases can regulate nucleotide enzymes directly. ALK has been shown to phosphorylate GUK1 and IMPDH2 [18,82], linking oncogenic kinase signaling to both downstream guanine nucleotide interconversion and de novo guanine nucleotide synthesis. This provides a mechanistic basis by which kinase inhibition could perturb guanine nucleotide balance while simultaneously creating selective pressure for compensatory nucleotide pathways.

The RAS-MAPK pathway similarly regulates both purine and pyrimidine metabolism. ERK-mediated phosphorylation of CAD and PFAS promotes pyrimidine and purine biosynthesis, respectively [80,83] and ERK inhibition reduces purine metabolites including IMP, AMP, and GMP. Oncogenic KRAS can reinforce this metabolic output by increasing nonoxidative PPP flux and ribose-5-phosphate production while engaging MAPK- and MYC-dependent transcriptional programs that support proliferation [84]. Downstream alterations in MEK can sustain ERK signaling and its associated metabolic programs [85], while BRAF mutations can activate the pathway independently of RAS [86]. Thus, nucleotide production represents an important metabolic output of MAPK signaling across multiple oncogenic contexts.

The emergence of next-generation KRAS inhibitors, including daraxonrasib, with activity in pancreatic and lung cancers further increases the relevance of these adaptive responses [87]. As increasingly potent inhibitors achieve deeper and more sustained suppression of RAS signaling, residual tumor cells may become more dependent on metabolic pathways that preserve nucleotide homeostasis. Defining these treatment-induced metabolic dependencies will be important for distinguishing mechanisms that merely accompany pathway inhibition from those that functionally enable persistence and acquired resistance.

One prominent adaptation to MAPK inhibition is a shift from de novo nucleotide synthesis toward salvage. MAPK pathway inhibition reduces both purine and pyrimidine production [80,83], while tumor cells can compensate by increasing uptake and utilization of extracellular nucleosides [88]. Stress-response signaling, particularly ATF4 activation, further supports this adaptation by promoting amino acid metabolism and one-carbon flux needed to maintain nucleotide homeostasis [89]. This ability to switch between de novo synthesis and salvage illustrates the metabolic plasticity that can blunt the impact of targeted oncogene inhibition. It also raises the possibility that salvage-pathway dependencies, including downstream guanine nucleotide enzymes such as GUK1, may become selectively actionable in the drug-treated state.

### 4.2. Targeted Therapy-Induced Metabolic Compensation

Targeted therapy can also induce metabolic compensation that indirectly sustains nucleotide synthesis. EGFR-altered lung adenocarcinomas exhibit extensive metabolic reprogramming [90], with oncogenic EGFR promoting aerobic glycolysis, PPP flux, and nucleotide biosynthesis through pathways that include mTOR signaling and increased glucose transporter expression [91]. EGFR inhibition reverses several of these features in TKI-sensitive cells, but incomplete suppression or subsequent metabolic adaptation can preserve access to the carbon and nitrogen substrates required for nucleotide production. These observations emphasize that nucleotide homeostasis is embedded within a larger metabolic network rather than controlled by nucleotide enzymes in isolation.

This metabolic interdependence creates opportunities for rational combination therapy. Simultaneous inhibition of EGFR with erlotinib and glutamine metabolism with the glutaminase inhibitor CB-839 markedly reduces tumor growth in both *in vitro* and *in vivo* models [92]. Such approaches are particularly relevant because glutamine supplies both nitrogen and carbon to nucleotide biosynthesis, providing a route by which tumor cells may buffer the metabolic consequences of oncogene inhibition. Beyond its effects on central carbon metabolism, EGFR signaling further contributes to lipid metabolic regulation, including modulation of enzymes such as stearoyl-CoA desaturase-1 (SCD1) [93], thus underscoring the metabolic impact of this pathway.

Metabolic compensation is also evident in ALK-rearranged NSCLC. Although nucleotide-specific adaptations remain less completely defined than in EGFR- or MAPK-driven models, ALK-positive tumors exhibit high glucose metabolic activity relative to EGFR-mutant tumors by FDG-PET/CT [94]. Oncogenic ALK can transcriptionally upregulate PFKFB3, a key regulator of glycolytic flux, and ALK and PFKFB3 expression correlate in clinical ALK-positive NSCLC specimens. Notably, both wild-type and TKI-resistant ALK-expressing cells remain sensitive to pharmacologic PFKFB3 inhibition [95]. Also, prolonged crizotinib exposure can further increase glycolytic dependence through HK2 upregulation in ALK-positive NSCLC, while glycolytic inhibition with 2-deoxy-D-glucose (2DG) suppresses proliferation, colony formation, invasion, and glycolytic flux in resistant cells [96]. These findings suggest that metabolic dependencies established by the oncogenic driver may persist after kinase resistance emerges and could provide orthogonal therapeutic vulnerabilities.

However, an unresolved question is which of these upstream metabolic changes are required to maintain nucleotide pools in resistant tumors and therefore which represent *bona fide* resistance dependencies rather than associated phenotypes. Importantly, many of the studies defining these metabolic adaptations were performed using earlier generations of targeted therapies, which often achieved less complete or less selective suppression of the oncogenic driver. As increasingly potent, selective, and durable targeted therapies enter clinical practice, it remains unclear whether resistant cells engage the same compensatory metabolic programs or whether deeper pathway suppression creates qualitatively distinct metabolic states and dependencies. Defining how the magnitude, selectivity, and duration of oncogene inhibition shape nucleotide metabolic rewiring will therefore be important for identifying vulnerabilities that remain relevant across successive generations of targeted therapy.

### 4.3. Tumor Suppressor Loss as a Modifier of Nucleotide Stress Tolerance

The metabolic response to targeted therapy is strongly influenced by co-occurring tumor suppressor alterations. In KRAS-mutant NSCLC, loss of STK11/LKB1 and/or KEAP1-NRF2 pathway dysregulation defines clinically challenging subsets associated with resistance to multiple therapies [97,98]. These alterations reprogram nutrient utilization, redox homeostasis, and biosynthetic demand, potentially changing how tumors respond to the nucleotide stress imposed by oncogene inhibition. Rather than representing a uniform metabolic state, KRAS-driven tumors therefore contain genetically defined subgroups with distinct dependencies that may influence both primary sensitivity and the routes available for acquired resistance.

A prominent feature of KEAP1/NRF2-altered tumors is increased reliance on glutamine metabolism. These tumors show sensitivity to glutaminase (GLS) inhibition, and KEAP1 loss further enhances this dependency through NRF2-driven metabolic rewiring [99]. As glutamine is incorporated directly into both purine and pyrimidine synthesis, this phenotype provides a mechanistic link between tumor suppressor status and the capacity to maintain nucleotide production under therapeutic stress.

Pillai et al. further demonstrated that KEAP1-mutant tumors depend on glutamine to sustain NRF2-mediated antioxidant and biosynthetic programs and that the glutamine-targeting prodrug DRP-104 suppresses tumor growth in KEAP1-mutant models [100]. Mechanistically, this activity is attributable in part to disruption of glutamine-dependent nucleotide biosynthesis. These data suggest that genotype-specific nutrient dependencies can constrain the metabolic routes available to resistant cells and may create opportunities to combine oncogene-directed therapy with inhibition of nucleotide precursor supply.

Concurrent STK11 and KEAP1 loss also produces broader metabolic and redox liabilities. Wohlhieter et al. identified a dependence on ferroptosis-protective mechanisms in co-mutant cells and identified SCD1 as an essential gene and potential therapeutic target [101]. Although this vulnerability is not nucleotide-specific, it illustrates how tumor suppressor genotype can reshape the stress-tolerance landscape in which nucleotide adaptation occurs and may influence which metabolic combinations are therapeutically feasible.

A more direct nucleotide dependency is seen in KRAS-mutant NSCLC with STK11/LKB1 loss. In this setting, KRAS-driven metabolic rewiring induces the urea-cycle enzyme CPS1, allowing tumor cells to channel mitochondrial ammonia into carbamoyl phosphate production and thereby support pyrimidine biosynthesis. Suppression of CPS1 produces pyrimidine depletion, replication stress, DNA damage, and impaired tumor growth [102]. This pathway demonstrates how a specific genomic context can create a noncanonical route for maintaining nucleotide supply and exposes a metabolic liability that could become especially consequential when oncogenic signaling is therapeutically suppressed.

Additional tumor suppressors, including PTEN, RB1, and ARID1A, can also influence nucleotide metabolism through effects on PI3K-AKT-mTOR signaling, cell-cycle regulation, and chromatin-dependent transcriptional programs, respectively, further illustrating how distinct genomic alterations may shape nucleotide metabolic dependencies. Across these molecular contexts, therapies do not always simply reduce nucleotide synthesis, but rather change the routes by which nucleotide pools are maintained. Tumor cells can compensate through salvage, altered precursor utilization, stress-response programs, or genotype-specific metabolic pathways, thus preserving the nucleotide balance required for survival and eventual regrowth. A central challenge for the field is to identify which adaptations are transient features of drug-tolerant persistence and which become stable dependencies in acquired resistance.

## 5. Nucleotide Metabolism in Immunotherapy Resistance

### 5.1. Nucleotide Competition and Shared Metabolic Dependencies in the Tumor-Immune Microenvironment

Historically, nucleotide metabolism in cancer has been studied primarily as a tumor-intrinsic biosynthetic program. More recent work supports a model in which nucleotide metabolism shapes reciprocal interactions between tumor cells and the immune compartment by controlling access to shared metabolic precursors, extracellular nucleotide signaling, and immune-cell fitness. A pan-cancer in silico analysis associated high nucleotide metabolic activity with low tumor microenvironment immune infiltration (TME-IF) and worse prognosis, whereas tumors with lower nucleotide activity and greater immune infiltration showed more favorable outcomes and treatment responsiveness [103]. While this is associative, these findings suggest that the metabolic state of a tumor and the composition of its immune microenvironment are linked and may jointly influence therapeutic response.

Competition for shared nutrients provides one mechanism connecting tumor nucleotide demand to impaired antitumor immunity. Tumor cells can preferentially consume metabolites required by infiltrating lymphocytes; Chang et al. demonstrated that tumor cells outcompete T cells for glucose in the TME, resulting in impaired T-cell effector function [104]. As glucose and amino acids also supply the pentose phosphate and one-carbon pathways that support nucleotide synthesis, competition for these precursors may simultaneously sustain tumor anabolic programs while limiting the proliferative and effector capacity of immune cells. Stromal cells can further reshape this metabolic environment by supplying alternative nutrients. For example, cancer-associated fibroblasts in pancreatic ductal adenocarcinoma secrete alanine that supports tumor biosynthesis under nutrient-limited conditions [105]. The extent to which stromal metabolite exchange directly alters nucleotide availability in tumor versus immune cells remains an area for investigation.

One-carbon metabolism illustrates the challenge of therapeutically separating tumor and immune nucleotide requirements. Cancer cells use serine-derived one-carbon units to sustain de novo purine synthesis, yet activated T cells rely on many of the same metabolites. Formate supplementation within the TME enhanced CD8+ T-cell activation and proliferation and improved tumor clearance when combined with immune checkpoint blockade. Notably, both tumor cells and CD8+ T cells were capable of utilizing formate to support proliferative programs [106]. These observations highlight that manipulating nucleotide precursor availability may either restrain tumor growth or support antitumor immunity depending on cell type, context, and timing.

A similar principle applies to pyrimidine metabolism. Dihydroorotate dehydrogenase (DHODH) inhibitors suppress de novo pyrimidine synthesis and are undergoing clinical investigation (NCT03404726), but systemic pathway inhibition can also constrain T-cell proliferation and function. Cytidine supplementation and targeting extracellular cytidine deaminase preserved effector T-cell function during DHODH inhibition in cancer models [107], suggesting that selective metabolic rescue of immune cells may widen the therapeutic window for nucleotide-directed therapy. A complementary example is provided by KEAP1-mutant tumors, in which the glutamine-targeting prodrug DRP-104 suppresses tumor growth by disrupting glutamine-dependent nucleotide biosynthesis while also enhancing antitumor T-cell responses [100]. This dual tumor-intrinsic and immune effect illustrates how metabolic interventions can potentially be designed to exploit differential nutrient dependencies across cellular compartments.

An additional therapeutic opportunity involves exploiting synthetic lethal vulnerabilities in MTAP-deleted tumors, where methylthioadenosine (MTA) accumulation creates selective sensitivity to PRMT5 and MAT2A inhibition. Emerging MTA-cooperative PRMT5 inhibitors have demonstrated encouraging early clinical activity across multiple solid tumors, highlighting opportunities to exploit tumor-specific nucleotide-associated metabolic vulnerabilities [108,109].

### 5.2. Extracellular Purine Signaling and the CD39-CD73-Adenosine Axis

Stress, hypoxia, and treatment-induced cell death in the TME can heavily remodel the extracellular nucleotide pool. Hypoxia restricts nutrient availability and leads to metabolic stress on both tumor and immune cells [104,110,111], promoting release of extracellular nucleotides such as ATP that signal through P2 receptors [112,113,114]. Cytotoxic therapy can further amplify this process, as immunogenic chemotherapies including anthracyclines and oxaliplatin can promote ATP release from dying tumor cells through mechanisms involving autophagy and pannexin-1 channels [115,116,117]. Extracellular ATP can initially function as a signal that promotes antitumor immunity, but its persistence and downstream metabolism determine whether this response remains immunostimulatory or becomes immunosuppressive.

CD39 and CD73 are central determinants of this balance by sequentially converting extracellular ATP-derived nucleotides to adenosine. Adenosine signals through P1 receptors in the TME to suppress cytotoxic T-cell and NK-cell activity and establish an immunosuppressive TME [118,119,120,121,122]. Inhibition of extracellular nucleotidases enhances cytotoxic T-cell responses and can sensitize resistant tumors to anti-PD1 immunotherapy *in vivo* [123]. Beyond pharmacologic inhibition of adenosine production, metabolic engineering of adoptive cellular therapies offers another strategy to overcome immunosuppression. CRISPR-mediated deletion of the adenosine A2A receptor in CAR-T cells enhances resistance to adenosine-mediated suppression and improves antitumor efficacy in preclinical models [124]. Clinical and preclinical observations further link this axis to treatment response: elevated CD73 expression has been associated with reduced responsiveness to anthracycline-based chemotherapy in triple-negative breast cancer, whereas pharmacologic CD73 inhibition can enhance oxaliplatin-induced antitumor immunity in colorectal cancer [125]. Therapy-induced extracellular ATP may also act directly on surviving tumor cells to promote pro-survival purinergic and HIF-1α signaling [53,126], emphasizing that the same extracellular nucleotide landscape can simultaneously shape tumor-cell persistence and immune escape.

### 5.3. cGAMP-STING Signaling and Cyclic Dinucleotide Exchange

The cyclic dinucleotide cGAMP provides a second example of how nucleotide metabolites coordinate communication between tumor and immune cells. ENPP1-mediated degradation of extracellular cGAMP promotes metastasis, reduces immune infiltration, and confers resistance to immunotherapy in tumor-bearing mice. Therapeutically, ENPP1 inhibition may provide dual immunologic benefits by preserving extracellular cGAMP to sustain STING signaling while limiting the generation of immunosuppressive adenosine, thereby enhancing antitumor immunity [127]. Conversely, micronuclei generated by radiation or chemotherapy exposure can stimulate cGAS-dependent cGAMP production and enhance STING signaling in activated CD4+ and CD8+ T cells, strengthening antitumor immunity [128]. cGAMP transport adds an additional layer of context dependence, as LRRC8C/LRRC8A transporters mediate cGAMP uptake in T cells, where STING-p53 signaling can suppress T-cell proliferation and effector function and deletion of LRRC8C produces enhanced antiviral phenotypes in mouse models [129]. Thus, cGAMP abundance, degradation, transport, and the identity of the responding cell collectively determine whether STING-pathway activation supports or restrains antitumor immunity. Recent work in small cell lung cancer further demonstrated that vascular STING activation can overcome spatial barriers to NK-cell infiltration and enhance antitumor cytotoxicity, providing a potential strategy to improve the efficacy of DLL3-targeted CAR-NK cellular therapies [130].

### 5.4. Nucleotide Cofactors and RNA-Linked Regulation of Immune-Cell Fitness

Nucleotide-derived cofactors can also influence the durability of antitumor immune responses. Nicotinamide adenine dinucleotide (NAD) availability declines in T cells with aging and has been linked to impaired memory T-cell differentiation and CAR-T exhaustion [131]. NAD precursor supplementation combined with inhibition of CD38-mediated NAD degradation restored antitumor activity in aged CAR-T cells [131], highlighting nucleotide cofactor homeostasis as another potential determinant of immune-cell persistence and therapeutic efficacy.

Post-transcriptional regulation of nucleotide-derived macromolecules may further contribute to immune escape. The RNA reader YTHDF2, which recognizes both m6A and m5C modifications, stabilizes m5C-containing ATP-synthesis transcripts through PABPC1 recruitment, promoting malignant B-cell survival and proliferation. In parallel, YTHDF2 destabilizes m6A-modified transcripts encoding CD19 and HLA-DMA/B, thereby facilitating immune escape from targeted immunotherapies [132]. These findings broaden the interface between nucleotide biology and treatment resistance beyond metabolite abundance alone to include nucleotide-dependent regulation of RNA stability and immune recognition.

Nucleotide metabolism thus represents a double-edged therapeutic axis in immuno-oncology. The same pathways that sustain tumor growth and adaptation are often required for lymphocyte activation, proliferation, and persistence, while extracellular nucleotide metabolites can either stimulate or suppress antitumor immunity depending on how they are processed and sensed. Future studies will need to define cell-type-specific metabolic dependencies, the spatial and temporal consequences of pathway inhibition, and whether tumor-directed nucleotide depletion can be paired with selective immune-cell rescue or blockade of immunosuppressive extracellular nucleotide metabolism. Such approaches may enable nucleotide-targeted therapies to overcome metabolically mediated immune resistance without compromising the antitumor immune response.

### 5.5. Spatial and Single-Cell Multiomics Reveal Metabolic Heterogeneity in the Tumor Microenvironment

Recent advances in single-cell and spatial technologies are providing new opportunities to investigate the spatial organization of metabolic reprogramming within the tumor microenvironment. Conventional bulk metabolomic approaches can obscure regional and cell-type-specific metabolic heterogeneity, particularly at interfaces between malignant cells, immune infiltrates, and stromal populations. Emerging platforms integrating spatial transcriptomics, multiplexed imaging, and mass spectrometry imaging-based metabolomics now enable the characterization of metabolic niches while preserving tissue architecture. Studies in gastric cancer and hepatocellular carcinoma have demonstrated spatially distinct metabolic programs at tumor-stromal and tumor-immune interfaces, highlighting the contributions of local nutrient availability and intercellular metabolic interactions to tumor biology [133,134]. Similarly, in breast cancer, spatial variations in oxygen and nutrient availability have been highlighted as important determinants of intratumoral metabolic heterogeneity, with distinct metabolic adaptations emerging across tumor regions depending on their proximity to vasculature and local microenvironmental conditions [135]. Furthermore, advances in single-cell spatial metabolomics coupled with proteomic profiling have enabled the identification of cell-type-specific metabolic states and local patterns consistent with metabolite competition [136,137]. Integrating spatial metabolomics with single-cell transcriptomic and proteomic analyses may therefore help identify tumor subpopulations with distinct metabolic adaptations, uncover spatially restricted mechanisms of therapeutic resistance, and inform the development of more precise metabolism-directed therapeutic strategies.

Taken all together, these immune-tumor metabolic mechanisms can be viewed through three complementary processes: competition for shared metabolic precursors, extracellular nucleotide-mediated immunomodulation, and regulation of immune-cell metabolic fitness, each of which may contribute to therapeutic resistance through distinct but interconnected pathways.

## 6. Radiation Therapy and Nucleotides

Radiation therapy remains a cornerstone of treatment for many solid tumors, yet radioresistance remains an important cause of local recurrence and treatment failure. Recurrence within previously irradiated high-dose fields underscores the contribution of tumor cell-intrinsic mechanisms that enable survival and recovery after radiation-induced stress [138].

Ionizing radiation induces a spectrum of DNA lesions, including DNA double-strand breaks, and tumor-cell survival is determined not only by the extent of initial damage but also by the capacity to enact repair pathways. DNA repair creates an acute demand for deoxynucleotides, directly linking radiation response to nucleotide availability. Early studies demonstrated induction of ribonucleotide reductase (RNR) after radiation, providing evidence that increased deoxynucleotide synthesis supports recovery from genotoxic stress [139]. Radiation can further enhance RNR activity through deacetylation of its RRM2 subunit, increasing nucleotide production required for DNA repair [140]. In parallel, nucleotide salvage pathways, including those involving deoxycytidine kinase, can replenish intracellular nucleotide pools and support DNA synthesis and repair, thereby contributing to radioresistance [141].

The temporal dynamics of nucleotide availability may also influence radiation response, as irradiation increases intracellular dCTP levels, while compensatory deoxynucleoside salvage can sustain DNA repair during RNR inhibition [142]. Importantly, dNTP availability also modulates DNA repair pathway choice, with RNR inhibition preferentially impairing homologous recombination-dependent repair [143]. These findings have implications for radiation fractionation and treatment scheduling, as preclinical studies demonstrate that RNR inhibition enhances radiosensitivity across different radiation dose rates, suggesting that the timing of nucleotide-directed therapies relative to radiation exposure may influence therapeutic efficacy [144].

Longer-term adaptation to radiation also involves metabolic rewiring that sustains nucleotide supply. Glutamine provides carbon and nitrogen for both purine and pyrimidine biosynthesis, linking glutamine metabolism to the nucleotide demands of DNA replication and repair. Perturbation of glutamine metabolism, including the knockdown of glutamine synthetase, sensitizes tumor cells to radiation [145]. Purine metabolism represents a complementary determinant of radiation response, with increased purine biosynthesis enhancing DNA repair capacity, whereas disruption of this pathway impairs recovery from radiation-induced damage and increases radiosensitivity [146]. In glioblastoma, Zhou et al. demonstrated that inhibition of IMPDH with mycophenolic acid (MPA) or its prodrug mycophenolate mofetil (MMF) lowers intracellular GTP levels, compromises DNA repair, and increases sensitivity to radiation, directly linking guanine nucleotide availability to radioresistance.

Nucleotide metabolic adaptation to radiation is also integrated with cellular redox homeostasis. Radioresistant cancer stem-like cells maintain low reactive oxygen species levels, thereby limiting radiation-induced damage [147]. Since glutamine metabolism contributes to both nucleotide production and redox control, its inhibition can restore radiosensitivity in selected settings. Functional genomic studies have further identified metabolic dependencies that support survival after radiation, including in KEAP1-mutant tumors. These observations raise the possibility that tumor genotype and redox state may define context-specific metabolic dependencies that can be exploited to enhance radiation response.

These data support a model in which nucleotide metabolism contributes to radioresistance across multiple stages of the radiation response: acutely, by replenishing nucleotide pools required for DNA repair, and over longer time scales, through metabolic programs that sustain nucleotide and redox homeostasis. RNR activity, glutamine metabolism, and purine biosynthesis represent candidate therapeutic vulnerabilities. Beyond conventional external beam radiotherapy, targeted radioligand therapies introduce additional opportunities to investigate whether prolonged radionuclide exposure and distinct radiation dose rates create specific nucleotide metabolic dependencies that could be exploited therapeutically [148]. Ongoing investigations are centered around defining biomarkers of these metabolic states and determining which tumors are most likely to benefit from combinations of radiotherapy and nucleotide-directed metabolic therapies.

## 7. Therapeutic Targeting of Nucleotide Metabolism: Opportunities and Challenges

### 7.1. Established and Emerging Nucleotide-Directed Therapies

Nucleotide metabolism is an attractive therapeutic target because malignant cells must continuously match nucleotide supply to the demands of proliferation, signaling, and stress adaptation. The longstanding clinical activity of antimetabolites validates this vulnerability but also illustrates an important limitation: nucleotide pathways are required by normal proliferating tissues and immune cells, constraining the therapeutic window of nonselective pathway inhibition. Current efforts are therefore shifting from broadly suppressing nucleotide synthesis toward identifying context-specific dependencies that emerge in defined tumor genotypes or under therapeutic stress.

Several clinically established therapies already disrupt pyrimidine or deoxynucleotide production (Table 1). Thymidylate synthase (TS) inhibitors, including 5-fluorouracil and pemetrexed, deplete thymidylate pools and induce replication stress, while ribonucleotide reductase (RNR) inhibitors such as hydroxyurea or gemcitabine restrict dNTP production [149,150]. Dihydroorotate dehydrogenase (DHODH), which couples de novo pyrimidine synthesis to mitochondrial respiration, has emerged as a complementary target, with agents including brequinar, leflunomide, and BAY2402234 having been evaluated in preclinical and early clinical studies [151,152,153,154]. These approaches may be most effective when treatment creates an acquired dependence on pyrimidine synthesis rather than when used as indiscriminate antiproliferative agents.

Purine metabolism provides additional opportunities to target nucleotide homeostasis downstream of convergent de novo and salvage inputs. IMPDH inhibition with mycophenolic acid or mycophenolate mofetil reduces guanine nucleotide and GTP availability and can perturb DNA/RNA synthesis as well as GTP-dependent signaling [156,157]. GMPS and GUK1 represent more distal nodes in guanine nucleotide metabolism and remain preclinical targets [18,158]. Targeting downstream nucleotide interconversion may be particularly attractive in resistant tumors because blockade of a single upstream biosynthetic route can be bypassed through salvage or extracellular metabolite uptake.

Salvage metabolism also influences response to nucleotide-directed therapy. Enzymes such as DCK and UCK1/2 determine the utilization of extracellular nucleosides and, in some settings, activation of nucleoside analog therapies. Thus, DCK activity has additionally been explored as a functional imaging and response biomarker [159,160,161,166,167]. Extracellular nucleotide metabolism represents a mechanistically distinct therapeutic axis. CD73 converts AMP to immunosuppressive adenosine, and CD73-directed agents are being developed largely in combination with immunotherapy, although compensatory adenosine-generating pathways may limit single-agent activity [164,165].

### 7.2. Exploiting Adaptive Nucleotide Dependencies in Resistant Tumors

As emphasized throughout this Review, therapeutic pressure can suppress one route of nucleotide production while increasing reliance on another, including shifts between de novo synthesis, salvage, and extracellular metabolite uptake. Consequently, the most actionable vulnerabilities may be induced by therapy rather than constitutively present in untreated tumors. This provides a rationale for treatment combinations in which oncogene-directed therapy, chemotherapy, radiation, or immunotherapy first creates a nucleotide dependency that is subsequently or concurrently targeted.

This framework also favors targeting regulatory nodes that integrate oncogenic signaling with nucleotide homeostasis rather than simply depleting nucleotides globally. Context-specific dependencies such as GUK1, IMPDH, DHODH, or salvage enzymes may offer greater selectivity when they become essential only after pathway inhibition or in defined molecular subsets. Conversely, simultaneous blockade of multiple redundant nucleotide routes could deepen tumor responses but may also increase toxicity in normal proliferating tissues. Determining which compensatory pathway is engaged in a given resistant state will therefore be critical for rational combination design.

### 7.3. Translational Challenges and Biomarker-Guided Development

Clinical translation of nucleotide-directed therapies will require strategies to distinguish tumor-selective metabolic dependencies from ubiquitous housekeeping functions. Static expression of a metabolic enzyme may not reliably identify pathway dependence because nucleotide demand is shaped by oncogenotype, nutrient availability, treatment history, and competing features in the tumor microenvironment. Genomic features can enrich for selected metabolic states, but functional biomarkers that capture pathway activity, nucleotide pool balance, or compensatory flux are likely to be necessary for patient selection.

Early clinical trials of nucleotide-directed therapies illustrate the challenges of establishing therapeutic windows. The DHODH inhibitor brequinar demonstrated dose-limiting thrombocytopenia and dermatologic toxicity, with mucositis also frequently observed, while the IMPDH inhibitor tiazofurin was limited by pleuropericarditis and systemic inflammatory-like symptoms [168,169]. In contrast, the CD73 inhibitor oleclumab demonstrated a more favorable tolerability profile without dose-limiting toxicities during dose escalation, although clinical activity was modest [162]. Clinical studies comparing weekly and biweekly brequinar administration further underscore the importance of treatment scheduling, with intermittent dosing potentially allowing normal tissue recovery while maintaining pathway inhibition [170]. Integrating optimized dosing schedules with tumor-selective metabolic dependencies, compensatory salvage activity, and pharmacodynamic biomarkers may help improve patient selection and expand therapeutic windows.

Pharmacodynamic approaches may include metabolite profiling, paired tumor sampling, functional imaging, or assays that measure pathway engagement before and after therapy. dCK-directed PET imaging provides one example of how enzyme activity rather than expression alone might be used to identify tumors capable of activating nucleoside analogs [159,160,161]. More broadly, defining when a metabolic dependency emerges, whether it persists during acquired resistance, and how it differs between tumor and immune compartments will be essential for establishing therapeutic windows. Future trials should therefore prioritize biomarker-anchored combinations and dynamic assessment of metabolic response rather than treating nucleotide metabolism as a uniform vulnerability across cancers.

Lastly, utilizing patient samples directly to interrogate metabolism is of the utmost importance. Formalin-fixed, paraffin-embedded (FFPE) tumor specimens, particularly archival tissues from clinically annotated patient cohorts, represent a valuable resource for investigating metabolic adaptations and therapeutic resistance. Although tissue fixation and processing can limit direct metabolomic analyses, spatial and single-cell technologies have expanded opportunities for metabolic profiling of FFPE specimens. These include spatial transcriptomics and single-cell proteomic approaches which enable assessment of metabolic enzyme expression and pathway-associated genes while preserving tissue architecture [171,172]. Such approaches may provide insights into nucleotide biosynthesis, salvage pathways, and metabolic heterogeneity across malignant, stromal, and immune populations. Furthermore, mass spectrometry imaging can be integrated with genomic and transcriptomic profiling of FFPE tissues to facilitate multimodal metabolic characterization, although limited by fixation-induced metabolite loss and chemical alterations that may compromise accurate assessment of native metabolic profiles [173]. These advances may enable identification of clinically relevant metabolic signatures associated with tumor genotype and therapeutic response, although profiling is an indirect measure of metabolic activity that requires functional validation.

## 8. Conclusions and Future Directions

Dysregulated nucleotide metabolism is a core hallmark of cancer cell biology across diverse lineages and thus represents a fundamental pan-cancer metabolic dependency. Beyond providing substrates for DNA and RNA synthesis, nucleotides regulate diverse cellular processes relevant to malignant growth and therapeutic adaptation, including GTPase signaling, nuclear transport, protein translation, redox homeostasis, and responses to metabolic and genotoxic stress. Accordingly, perturbing nucleotide homeostasis can have consequences that extend well beyond inhibition of proliferation. These broader functions underscore the therapeutic potential of targeting nucleotide metabolism, particularly in rational combination strategies designed to exploit dependencies that emerge under treatment pressure.

In this Review, we highlight nucleotide metabolism as a shared adaptive axis across multiple forms of therapeutic resistance. Chemotherapy, targeted therapies, immunotherapy, and radiation impose distinct metabolic stresses, yet resistant cells frequently converge on mechanisms that preserve or redistribute nucleotide pools through altered *de novo* synthesis, salvage, transport, and pathway-specific feedback. A central challenge is therefore to distinguish metabolic changes that merely accompany resistance from those that are functionally required to sustain the resistant state. Thus, genetic perturbations, metabolite rescues, *in vivo* validation, and temporally controlled models such as inducible knockouts or degron systems are needed to distinguish transient adaptations from mechanisms required for resistance. Genome-scale functional screening, metabolomics, isotope tracing, and perturbation-based approaches provide powerful tools to define these dependencies and to identify compensatory pathways that become essential after therapeutic stress. Importantly, metabolic state represents an additional dimension of tumor biology that is not fully captured by genomics, transcriptomics, or proteomics alone and may more directly reflect nutrient availability, pathway flux, and cellular demand.

Translating these insights into clinical benefit will require greater emphasis on patient-derived systems and longitudinal sampling. Analysis of primary tumors, matched pre- and post-treatment specimens, circulating biomarkers, and patient-derived models will be essential for defining how nucleotide metabolism evolves during therapy *in vivo*. Such studies should also account for the tumor microenvironment, where stromal and immune cells both compete for and contribute metabolites that influence nucleotide availability. This issue is particularly important for therapeutic strategies that target pathways shared by tumor and immune cells, where efficacy will depend on achieving a sufficient therapeutic window. Integration of metabolic phenotyping with tumor genotype, lineage, oncogenic driver, tumor suppressor status, treatment exposure, and microenvironmental context may ultimately enable more precise selection of tumors most likely to respond to nucleotide-directed therapies.

Continued drug development should yield more potent and selective inhibitors of nucleotide metabolic pathways, but the greatest opportunity may lie in identifying context-specific metabolic dependencies created by therapy itself. As targeted agents become increasingly potent and selective, it will be important to determine whether resistant tumors engage the same metabolic programs described with earlier-generation therapies or develop qualitatively different adaptive states. Defining these therapy-specific vulnerabilities may enable rational combinations that prevent metabolic compensation, delay resistance, or resensitize refractory tumors. More broadly, the principles emerging from cancer nucleotide metabolism may also have relevance beyond oncology, including immune, infectious, and neurodegenerative diseases in which nucleotide homeostasis is similarly coupled to cellular stress and adaptation.

## Figures and Tables

**Table 1:** Selected nucleotide-metabolic targets and representative therapeutic strategies.

Target/Pathway	Representative Agents	Therapeutic Rationale	Clinical Development
DHODH/de novo pyrimidine synthesis	Brequinar; leflunomide; BAY2402234	Depletes pyrimidines; may exploit therapy-induced pyrimidine dependence	Preclinical to early clinical [151,152,153,154]
TS/folate-dependent pyrimidine synthesis	5-FU/capecitabine; pemetrexed; raltitrexed	Depletes dTMP and induces replication stress	Established clinical therapies [149]
RNR/dNTP synthesis	Hydroxyurea; gemcitabine; triapine	Restricts deoxynucleotide supply and DNA replication/repair	Established and late-stage strategies [150,155]
IMPDH/guanine nucleotide synthesis	Mycophenolic acid; mycophenolate mofetil	Reduces guanine nucleotides/GTP, affecting biosynthesis, signaling, and repair	Repurposing/preclinical cancer studies [156,157]
GMPS/GUK1/guanine nucleotide homeostasis	Decoyinine (GMPS); no validated clinical GUK1 inhibitor	Targets distal guanine nucleotide production/interconversion and may limit pathway compensation	Preclinical [18,158]
Nucleoside salvage/analog activation	Cytarabine; azacitidine; decitabine; DCK/UCK-dependent salvage	Salvage enzymes support nucleoside utilization and determine activation of selected analogs	Established analog therapies; DCK biomarker/imaging studies [159,160,161]
CD73/extracellular nucleotide metabolism	Oleclumab; AB680	Blocks AMP-to-adenosine conversion and immunosuppressive purinergic signaling	Early clinical combinations, often with checkpoint blockade [162,163,164,165]

Abbreviations: 5-FU, 5-fluorouracil; AB680, CD73 small-molecule inhibitor; AML, acute myeloid leukemia; CD73, ecto-5′-nucleotidase; DCK, deoxycytidine kinase; DHODH, dihydroorotate dehydrogenase; dTMP, deoxythymidine monophosphate; dTTP, deoxythymidine triphosphate; FdUMP, fluorodeoxyuridine monophosphate; GMPS, guanosine monophosphate synthase; GMP, guanosine monophosphate; GTP, guanosine triphosphate; IMP, inosine monophosphate; IMPDH, inosine monophosphate dehydrogenase; MMF, mycophenolate mofetil; MPA, mycophenolic acid; RNR, ribonucleotide reductase; TS, thymidylate synthase; UCK1/2, uridine-cytidine kinase 1/2; XMP, xanthosine monophosphate.

## Data Availability

No new data were generated or analyzed in this study. Data sharing is not applicable.
